# Routine Vaccination Coverage — Worldwide, 2023

**DOI:** 10.15585/mmwr.mm7343a4

**Published:** 2024-10-31

**Authors:** Camille E. Jones, M. Carolina Danovaro-Holliday, George Mwinnyaa, Marta Gacic-Dobo, Lauren Francis, Jan Grevendonk, Yoann Nedelec, Aaron Wallace, Samir V. Sodha, Ciara Sugerman

**Affiliations:** ^1^Epidemic Intelligence Service, CDC; ^2^Global Immunization Division, Center for Global Health, CDC; ^3^Department of Immunization, Vaccines and Biologicals, World Health Organization, Geneva, Switzerland; ^4^Division of Data Analytics, Planning and Monitoring, UNICEF, New York, New York.

**Summary**

**What is already known about this topic?**

The COVID-19 pandemic interrupted health systems worldwide, negatively affecting immunization programs; recovery has been uneven.

**What is added by this report?**

During 2022–2023, global immunization coverage plateaued at 89% with the first dose and 84% with the third dose of diphtheria-tetanus-pertussis–containing vaccine and 83% with the first dose of measles-containing vaccine. Coverage with these vaccines remains lower than 2019 prepandemic levels. Countries with fragile, conflict-affected, and vulnerable settings experienced disproportionate challenges in reaching unvaccinated and incompletely vaccinated children.

**What are the implications for public health practice?**

Strategies for strengthening routine immunization, catch-up vaccination, and new and underutilized vaccine introductions can improve the overall breadth of protection and support countries’ prevention of vaccine-preventable disease outbreaks.

## Abstract

In 2020, the World Health Assembly endorsed the Immunization Agenda 2030 (IA2030), a 10-year strategy to reduce vaccine-preventable disease (VPD)–associated morbidity and mortality. IA2030 goals include improving equitable vaccination coverage, halving the number of unimmunized (zero-dose) children, and increasing the introduction of new and underutilized vaccines. The COVID-19 pandemic disrupted health systems worldwide, hindering years of childhood vaccination achievements and putting global public health goals at risk. This report presents trends in World Health Organization (WHO) and UNICEF routine vaccination coverage estimates through 2023 across the 194 WHO member countries. During 2022–2023, global coverage with the first and third doses of diphtheria-tetanus-pertussis–containing vaccine (DTPcv) (89% and 84%, respectively) and the first dose of measles-containing vaccine (83%) stagnated and remained lower than prepandemic levels. The 31 WHO member countries with fragile, conflict-affected, and vulnerable (FCV) settings include approximately one half of the world’s 14.5 million children who did not receive the first DTPcv dose. The introduction of new and underutilized vaccines, such as a second MCV dose in the African Region, has improved countries’ overall protection against VPDs. Accelerating country-specific routine immunization and catch-up vaccination programs to reach unvaccinated and incompletely vaccinated children, especially those living in FCV settings, is critical to reducing morbidity and mortality associated with VPDs.

## Introduction

In 1974, the World Health Organization (WHO) launched the Expanded Programme on Immunization, which focused on delivering vaccines during the first year of life to protect every child against diphtheria, tetanus, pertussis, poliomyelitis, measles, and tuberculosis (*1*,*2*). Now referred to as the Essential Programme on Immunization, the program includes many additional antigens and vaccine doses recommended through childhood and adolescence.* In 2020, the World Health Ass embly endorsed the Immunization Agenda 2030 (IA2030), the 2021–2030 overarching global vision and strategy to reduce morbidity and mortality from vaccine-preventable diseases (VPDs) across the life course and leave no one behind (*3*). Goals of this strategy include improving equitable vaccination coverage, halving the number of zero-dose children (children who have not received the first dose of a diphtheria-tetanus-pertussis–containing vaccine [DTPcv]), and increasing introductions of new and underutilized vaccines. Progress was adversely affected by the COVID-19 pandemic, as impeded access to health services resulted in global declines in immunization coverage and a nearly 40% increase in the number of zero-dose children (*4*), increasing immunity gaps worldwide. This report updates a previous report (*5*) and presents global, regional,^†^ and national routine vaccination^§^ coverage trends during 2010–2023 across the 194 WHO member countries, highlighting trends before and during the COVID-19 pandemic, and through 2023.

## Methods

### Vaccination Coverage Estimates

WHO and UNICEF Estimates of National Immunization Coverage (WUENIC) are produced annually at national, regional, and global levels by reviews of country-level data, including administrative and survey-based coverage^¶^ (*6*,*7*). Data on vaccines routinely provided by national immunization programs are reviewed. Coverage estimates are produced for vaccines recommended during the first year of life, including Bacille Calmette-Guérin (BCG), first and third doses of DTPcv (DTPcv1 and DTPcv3), hepatitis B birth dose (HepB-BD) and third dose (HepB3), first dose of measles-containing vaccine (MCV1), rubella-containing vaccine (RCV), rotavirus vaccine last dose, first and second doses of inactivated poliovirus vaccine (IPV1 and IPV2), third dose of *Haemophilus influenzae* type b vaccine (Hib3), third dose of pneumococcal conjugate vaccine (PCV3), and third dose of polio vaccine (Pol3),** as well as yellow fever and meningococcal A vaccines in at-risk countries. Data on vaccines administered beyond the first year of life are also reviewed, including second dose of MCV (MCV2) and first and last doses of human papillomavirus vaccine (HPV, first and HPV, last).^††^

### Indicators of Program Performance and Service Utilization

Children who did not receive DTPcv1 are considered zero-dose children, reflecting poor access to immunization and other health services (*3*). Children who receive DTPcv1 but not DTPcv3 are considered incompletely vaccinated.^§§^ DTPcv3 coverage by age 12 months is a historical indicator of routine immunization program performance, and dropout before completing the DTPcv series or from DTPcv1 to MCV1 reflects underuse of services among children with access and a lack of continuity in primary health care services. Trends in DTPcv and MCV were assessed from 2010 to 2023 across WHO region and World Bank economic classification,^¶¶^ and were evaluated among countries with fragile, conflict-affected, or vulnerable (FCV) settings.*** This activity was reviewed by CDC, deemed not research, and was conducted consistent with applicable federal law and CDC policy.^†††^

## Results

### Diphtheria-Tetanus-Pertussis–Containing Vaccines

During 2010–2019, global coverage with DTPcvs remained relatively unchanged: DTPcv1 coverage was 89% in 2010 and 90% in 2019; DTPcv3 coverage increased from 83% in 2010 to 86% in 2019 (Table). During the COVID-19 pandemic, global coverage with DTPcv1 and DTPcv3 declined, reaching 86% and 81%, respectively, by 2021. By 2023, coverage had partially recovered (DTPcv1 = 89%; DTPcv3 = 84%) but still had not reached the 2019 prepandemic levels. By 2023, the Region of the Americas was the only region in which coverage was improved compared with 2019 (DTPcv1 increased to 91% from 89% in 2019; DTPcv3 increased to 86% from 84% in 2019).

**TABLE:** Coverage with first and third doses of diphtheria-tetanus-pertussis–containing vaccine and measles-containing vaccine by World Health Organization Region, World Bank economic classification, and World Bank–defined fragile, conflict-affected, and vulnerable settings — worldwide, 2010 and 2019–2023

Year/Vaccine	Global	Vaccination coverage (%)										
		WHO region*						Income classification^†^				Countries with FCV settings
		AFR	AMR	EMR	EUR	SEAR	WPR	Low	Lower-middle	Upper-middle	High	
**2010**												
DTPcv1	**89**	80	97	83	96	88	97	81	84	96	98	79
DTPcv3	**83**	71	94	74	94	82	96	72	77	94	96	71
MCV1	**84**	72	93	76	93	83	97	72	79	93	94	71
MCV2	**41**	4	67	51	80	15	87	8	13	85	83	13
**2019**												
DTPcv1	**90**	83	89	88	98	94	97	82	90	94	97	81
DTPcv3	**86**	77	84	84	95	91	96	75	86	91	95	75
MCV1	**86**	71	87	82	96	94	96	70	86	93	94	70
MCV2	**71**	33	72	75	92	83	93	34	69	85	93	39
**2020**												
DTPcv1	**88**	82	88	86	97	88	96	81	86	92	97	80
DTPcv3	**83**	74	81	80	94	86	95	73	82	88	94	72
MCV1	**83**	69	86	82	94	88	95	68	83	90	94	69
MCV2	**71**	39	73	75	91	80	93	35	71	84	92	46
**2021**												
DTPcv1	**86**	81	87	87	97	86	94	78	85	90	97	77
DTPcv3	**81**	73	81	80	94	83	93	69	81	86	95	69
MCV1	**81**	67	85	80	95	87	92	64	81	89	94	66
MCV2	**71**	40	77	75	91	79	91	35	71	84	92	45
**2022**												
DTPcv1	**89**	81	90	87	97	94	96	78	90	94	98	78
DTPcv3	**84**	73	83	81	95	92	95	68	86	91	95	69
MCV1	**83**	68	84	80	94	94	93	63	85	92	94	66
MCV2	**73**	44	76	75	91	86	92	37	75	86	92	46
**2023**												
DTPcv1	**89**	83	91	85	97	92	94	78	89	93	97	78
DTPcv3	**84**	74	86	79	95	90	92	68	85	90	94	70
MCV1	**83**	70	85	79	95	91	92	64	85	90	94	67
MCV2	**74**	49	75	73	91	85	90	42	77	82	91	50

**Abbreviations:** AFR = African Region; AMR = Region of the Americas; DTPcv = diphtheria-tetanus-pertussis–containing vaccine; DTPcv1 = first DTPcv dose; DTPcv3 = third DTPcv dose; EMR = Eastern Mediterranean Region; EUR = European Region; FCV = fragile, conflict-affected, and vulnerable; GNI = gross national income; MCV1 = first measles-containing vaccine dose; MCV2 = second measles-containing vaccine dose; SEAR = South-East Asia Region; WHO = World Health Organization; WPR = Western Pacific Region.

* Countries included are WHO member countries.

^†^ Economic classification is based on GNI per capita, calculated in U.S. dollars (https://datahelpdesk.worldbank.org/knowledgebase/articles/906519-world-bank-country-and-lending-groups). For the current 2025 fiscal year, low-income economies are defined as those with a GNI per capita, calculated using the World Bank Atlas method, of ≤$1,145 in 2023; lower-middle–income economies are those with a GNI per capita of $1,146–$4,515; upper-middle–income economies are those with a GNI per capita of $4,516–$14,005; high-income economies are those with a GNI per capita of >$14,005.

From 2019 to 2023, DTPcv coverage decreased in countries in every World Bank economic classification. The decline was most notable in low-income countries, where DTPcv3 decreased by 7 percentage points (75% in 2019 to 68% in 2023); countries in all other income groups returned to within 1 percentage point of 2019 coverage levels.

Before the COVID-19 pandemic, the global number of zero-dose children decreased 19%, from 15.8 million in 2010 to 12.8 million in 2019. In 2023, a total of 14.5 million children worldwide had not received DTPcv1. The 10 countries with the largest numbers of zero-dose children included, in descending order, Nigeria (2,134,000), India (1,592,000), Ethiopia (917,000), Democratic Republic of the Congo (839,000), Sudan (701,000), Indonesia (662,000), Yemen (580,000), Afghanistan (467,000), Angola (411,000), and Pakistan (396,000); together, these 10 countries accounted for 59% of the world’s zero-dose children.

**FIGURE 1:**
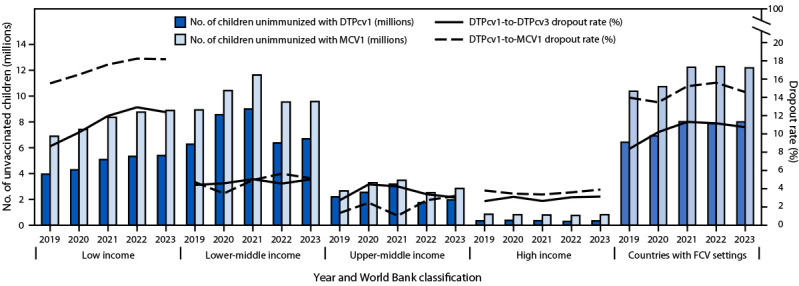
Estimated number of children who did not receive the first doses of diphtheria-tetanus-pertussis-containing vaccine and measles-containing vaccine, and dropout from the first to third dose of diphtheria-tetanus-pertussis–containing vaccine and from the first dose of diphtheria-tetanus-pertussis–containing vaccine to the first dose of measles-containing vaccine, by World Bank economic classification* and World Bank-defined fragile, conflict-affected, and vulnerable settings^†^ — worldwide, 2019–2023 **Abbreviations:** DTPcv = diphtheria-tetanus-pertussis–containing vaccine; DTPcv1 = first DTPcv dose; DTPcv3 = third DTPcv dose; FCV = fragile, conflict-affected, and vulnerable; GNI = gross national income; MCV1 = first measles-containing vaccine dose. * Economic classification is based on GNI per capita, calculated in U.S. dollars (https://datahelpdesk.worldbank.org/knowledgebase/articles/906519-world-bank-country-and-lending-groups). For the current 2025 fiscal year, low-income economies are defined as those with a GNI per capita, calculated using the World Bank Atlas method, of ≤$1,145 in 2023; lower-middle–income economies are those with a GNI per capita of $1,146–$4,515; upper-middle–income economies are those with a GNI per capita of $4,516–$14,005; high-income economies are those with a GNI per capita of >$14,005. ^†^ FCV is a broad term describing a range of situations, including humanitarian crises, protracted emergencies, and armed conflicts. The 31 countries listed as FCV in 2023 included Afghanistan, Bangladesh, Burkina Faso, Burma, Burundi, Cameroon, Central African Republic, Chad, Colombia, Democratic Republic of the Congo, El Salvador, Ethiopia, Guatemala, Haiti, Honduras, Iraq, Lebanon, Libya, Madagascar, Mali, Mozambique, Niger, Nigeria, Somalia, South Sudan, the Palestinian Territories, Sudan, Syria, Ukraine, Venezuela, and Yemen. https://gho.unocha.org/

The global DTPcv1-to-DTPcv3 dropout rate^§§§^ increased from 5% in 2019 to 6% in 2021 and remained at 6% in 2023. However, the dropout rate in low-income countries was higher than that in all other income levels and increased from 9% in 2019 to 13% in 2023 (Figure 1).

### Measles-Containing Vaccines

Globally, MCV1 coverage increased from 84% in 2010 to 86% in 2019 (Table). During the COVID-19 pandemic, MCV1 coverage declined to 81% in 2021, then partially recovered, reaching 83% in 2023. From 2010 to 2019, 42 countries introduced MCV2 into their immunization schedules,^¶¶¶^ and total global MCV2 coverage increased from 41% in 2010 to 71% in 2019. Most MCV2 introductions occurred in the African Region (26 countries), resulting in a regional increase in MCV2 coverage from 4% in 2010 to 33% in 2019. During 2020–2023, 10 additional countries in the African Region introduced MCV2, and regional coverage increased to 49% in 2023.

**FIGURE 2:**
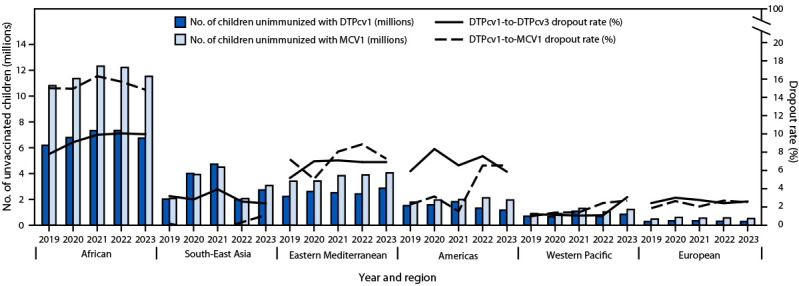
Estimated number of children who did not receive the first doses of diphtheria-tetanus-pertussis–containing vaccine and measles-containing vaccine, and dropout from the first to third dose of diphtheria-tetanus-pertussis–containing vaccine and from the first dose of diphtheria-tetanus-pertussis–containing vaccine to the first dose of measles-containing vaccine, by World Health Organization region* — worldwide, 2019–2023 **Abbreviations:** DTPcv = diphtheria-tetanus-pertussis–containing vaccine; DTPcv1 = first DTPcv dose; DTPcv3 = third DTPcv dose; MCV1 = first measles-containing vaccine dose. * Based on World Health Organization regional classifications. https://www.who.int/about/who-we-are/regional-offices

From 2019 to 2023, the number of children who were not immunized against measles worldwide increased 15%, from 19.3 million to 22.2 million. Global DTPcv1-to-MCV1 dropout rate**** decreased from 6% in 2010 to 5% in 2019, then increased to 7% in 2023. In the African Region, the DTPcv1-to-MCV1 dropout rate increased from 10% in 2010 to 15% in 2019 and remained unchanged in 2023 (Figure 2). Among low-income countries, DTPcv1-to-MCV1 dropout increased from 10% in 2010 to 16% in 2019 and to 18% in 2023 (Figure 1).

### Other Vaccines

From 2019 to 2021, global coverage with the following vaccines decreased: BCG (from 89% to 85%); HepB birth dose and third dose HepB (from 43% to 41%, and from 86% to 81%, respectively); Hib3 (from 74% to 72%); IPV1 (from 83% to 80%); Pol3 (from 83% to 81%); and RCV (from 69% to 66%) (Supplementary Table, https://stacks.cdc.gov/view/cdc/166709). In 2023, global IPV1 coverage returned to its 2019 level (83%). Despite an interruption in vaccine introductions in 2020 (*8*), many vaccine introductions took place during 2019–2023, including HepB-BD (nine countries) and rotavirus vaccine (17 countries). IPV2 was introduced in 58 oral poliovirus vaccine–using countries during 2019–2023; by 2023, global coverage with IPV2 was 42% (Table). HPV vaccination was introduced in 73 countries during 2010–2019 and 30 countries during 2020–2023. Global first-dose HPV coverage (HPV, first) among girls increased from 17% in 2019 to 27% in 2023, and full HPV coverage (HPV, last) from 13% in 2019 to 20% in 2023.

### Countries with Fragile, Conflict-Affected, and Vulnerable Settings

In 2023, the United Nations Office for the Coordination of Humanitarian Affairs defined 31 WHO countries as having FCV settings, representing a range of situations, including humanitarian crises, protracted emergencies, and armed conflicts. Among these countries, DTPcv1 and DTPcv3 coverage declined from 81% and 75%, respectively, in 2019 to 78% and 70%, respectively, in 2023 (Table). In countries with FCV settings, MCV1 coverage declined from 70% in 2019 to 66% in 2021 and 2022 and to 67% in 2023. By 2023, 59% of children who did not receive DTPcv1 and 55% of those who did not receive MCV1 lived in FCV countries. The number of zero-dose children increased by 25%, from 6.4 million in 2019 to 8.0 million in 2021 and then remained stagnant through 2023 (Figure 1). Similarly, DTPcv1-to-DTPcv3 dropout increased from 8% in 2019 to 11% in 2021 and remained at 11% in 2023. The number of children who were not immunized against measles increased 17%, from 10.4 million in 2019 to 12.2 million in 2021 and remained unchanged in 2023. DTPcv1-to-MCV1 dropout increased from 14% in 2019 to 15% in 2023; in the 163 non-FCV countries, the DTPcv1-to-DTPcv3 and DTPcv1-to-MCV1 dropout rates in 2023 were each only 4%.

## Discussion

After the COVID-19 pandemic and the resulting worldwide disruptions of health systems, countries that have been faced with declines in childhood immunization coverage have not yet recovered; these countries are, therefore, susceptible to higher VPD incidences. Most unimmunized children in 2023 lived in 31 countries with FCV settings.

Strategies such as periodic intensification of routine immunization and routine catch-up vaccination activities will be required to build resilience and mitigate impacts to the health system and are critical components of the ongoing Big Catch-Up strategy for immunization recovery (*4*). This strategy includes catching up children who missed vaccination during 2019–2022, restoring vaccination coverage rates for the current birth cohort to at least 2019 levels, and strengthening primary health care immunization systems. Country-level planning tailored to local contexts is needed, particularly within the varying conditions of humanitarian crises, protracted emergencies, and armed conflicts defining FCV settings.

Although coverage with well-established vaccines, such as DTPcv, stagnated before the COVID-19 pandemic and has been slow to return to prepandemic levels, new vaccine introductions have improved the mean coverage with all WHO-recommended vaccine antigens.^††††^ National and subnational introduction of new and underutilized vaccines helps to strengthen the impact of routine immunization programs and supports the IA2030 priority objective of strengthening immunization policies and service delivery throughout life (*3*).

### Limitations

The findings in this report are subject to at least six limitations. First, because the COVID-19 pandemic disrupted survey implementation, estimates in some countries are less informed by survey data than they were in years before the pandemic. Second, data quality limitations in some countries might have resulted in inaccurate administrative coverage estimations (*7*). Third, selection and recall bias might affect survey-based coverage estimates (*7*). Fourth, inaccuracies in population estimates could result in inaccurate estimates of zero-dose and incompletely vaccinated children. Fifth, coverage estimates do not include statistical uncertainty. Finally, although the WUENIC estimates characterize coverage among eligible cohorts vaccinated each year, data do not correct for doses administered late (for example, through catch-up vaccination activities, including those part of the Big Catch-Up global initiative) (*4*).

### Implications for Public Health Practice

Since the launch of the Expanded Programme on Immunization, much progress has been made in increasing immunization coverage; however, the COVID-19 pandemic-associated disruptions in health systems augmented existing gaps and introduced new gaps in immunization coverage worldwide. Progress toward recovery has been uneven across countries, with low-income countries, the African Region, and FCV settings having the most urgent need for action. Locally driven, country-specific strategies are needed to effectively strengthen routine immunization systems and provide catch-up vaccination to unvaccinated and incompletely vaccinated children to reduce the morbidity and mortality associated with VPDs worldwide.

## Footnotes

* WHO recommends additional vaccines based on region or population risk group. https://www.who.int/publications/m/item/table1-summary-of-who-position-papers-recommendations-for-routine-immunization

†
https://www.who.int/about/who-we-are/regional-offices

§ Routine vaccination refers to the part of the health system that facilitates vaccination service delivery to all eligible persons. This term also describes the process to regularly deliver vaccines according to the national vaccination schedule. https://iris.who.int/bitstream/handle/10665/204500/9789241510103_eng.pdf?sequence%20=%201

¶ Administrative coverage is calculated as doses administered during routine immunization visits within a specified target group divided by the estimated target population. Survey-based coverage is calculated as the proportion of persons in a target age group who received a vaccine dose. During surveys, a representative sample of households is visited, and caregivers of children in a specified target age group are interviewed. Vaccination dates are transcribed from home-based or health facility records. When documented evidence was unavailable, dates were recorded based on caregiver recall.

** Pol3 is defined as the percentage of surviving infants who received the third dose of a polio-containing vaccine. Doses may be administered as either oral poliovirus vaccine or IPV. https://www.who.int/teams/immunization-vaccines-and-biologicals/immunization-analysis-and-insights/global-monitoring/immunization-coverage/who-unicef-estimates-of-national-immunization-coverage

†† Vaccines provided during the first year of life versus after the first year of life are based on WHO routine immunization schedule recommendations (https://cdn.who.int/media/docs/default-source/immunization/immunization_schedules/table_1_april_2024_english.pdf?sfvrsn%20=%202e112cea_2&download%20=%20true). The age at which a specific vaccine is delivered might vary among different country routine immunization schedules based on the country-specific disease incidence associated with each vaccine.

§§ Incompletely vaccinated children might also be referred to as undervaccinated children.

*** FCV is a broad term describing settings experiencing a range of situations, including humanitarian crises, protracted emergencies, and armed conflicts. The 31 countries listed as FCV in 2023 included Afghanistan, Bangladesh, Burkina Faso, Burma, Burundi, Cameroon, Central African Republic, Chad, Colombia, Democratic Republic of the Congo, El Salvador, Ethiopia, Guatemala, Haiti, Honduras, Iraq, Lebanon, Libya, Madagascar, Mali, Mozambique, Niger, Nigeria, Somalia, South Sudan, the Palestinian Territories, Sudan, Syria, Ukraine, Venezuela, and Yemen. https://gho.unocha.org/

††† 45 C.F.R. part 46.102(l)(2), 21 C.F.R. part 56; 42 U.S.C. Sect. 241(d); 5 U.S.C. Sect. 552a; 44 U.S.C. Sect. 3501 et seq.

§§§ DTPcv1-to-DTPcv3 dropout rate = [(number of children vaccinated DTPcv1) – (number of children vaccinated DTPcv3)] / (number of children vaccinated DTPcv1) x 100%.

¶¶¶ https://immunizationdata.who.int/global/wiise-detail-page/vaccination-schedule-for-country_name

**** DTPcv1-to-MCV1 dropout rate = [(number of children vaccinated DTPcv1) – (number of children vaccinated MCV1)] / (number of children vaccinated DTPcv1) x 100%.

†††† Breadth of protection is defined as the mean coverage for all WHO-recommended vaccine antigens by country. The numerator is the sum of the coverage for the following antigens at the level of interest: diphtheria, tetanus, pertussis, hepatitis B, Hib, MCV1, MCV2, pneumococcus, oral poliovirus vaccine, IPV, rubella, rotavirus, and HPV. The denominator is the number of all antigens included for the indicator calculation, regardless of whether the antigen has been introduced at the level of interest (denominator equals 13). https://www.who.int/data/gho/indicator-metadata-registry/imr-details/7771
